# Society Meetings

**Published:** 1917-07

**Authors:** 


### SOCIETY MEETINGS

Brief reports and announcements of meetings of societies of Western New York. and those of general scope, are requested from Secretaries. Copy should be on hand the fifteenth of the month. Full transactions will be published at cost of composition.

---

**Elmira Academy of Medicine.** Regular meeting at Elmira, June 20. Cardio-therapy of the Obese, C. W. Lieb; title to be announced, A. W. Booth. R. B. Howland, M. D., President; O. J. Bowman, M. D., Secretary.

---

**Buffalo Academy of Medicine.** May 23, Section of Pathology, Some Modifications and Controls of the Wassermann Reaction, A. A. Thibaudeau; Research on Regeneration of Bone in Fractures, Roland O. Meisenbach.

June 13, the 25th anniversary of the establishment of the Academy was celebrated by a banquet at the Lafayette Hotel, the President, Dr. Lawrence Hendee, acting as toastmaster and showing stereopticon views of the early physicians and landmarks of Buffalo. Major J. W. Grosvenor, President 1903-4, in a brief address, presented evidence that he had been incorrectly marked as dead by the printer. Dr. DeLancey Rochester reviewed the history of the earlier organizations of the profession of Buffalo and of the steps taken to amalgamate them as sections of a single Academy. Dr. John H. Pryor spoke on the Aims and Objects of the Academy and Hon. Charles B. Sears on Patriotism.

During the meeting, the election of officers for the ensuing year took place, the following ticket, without opposition, being elected: Pres. Irving M. Snow; Sec. Herbert A. Smith; Treas. Benjamin F. Schreiner; Trustee for one year, Earl P. Lothrop.

The various reports were presented in printed form and have been distributed to the members. The Section Officers for the coming year are: Section of Surgery, J. B. Cross, Chairman, J. P. Brennan, Sec.; Section of Medicine, DeWitt Sherman Chairman, Jacob Otto Sec.; Obstetrics and Gynaecology, Herman K. De Groat Chairman, Edward E. Meister Sec.; Pathology, Wm. G. Bissell Chairman, Karl Eschelman Sec.

---

**The Alumni Association of the Medical Dept., University of Buffalo,** (organized 1875), held its 42d annual meeting May 31-June 2, independently of commencement on account of conflict with the meeting of the A. M. A. in N. Y. The officers of the meeting were as follows: Walter D. Greene, '76, President, Buffalo, N. Y. William H. Bergtold, '86, 1st Vice-Pres., Denver, Colo. Henry A. Eastman, '92, 2d Vice-Pres., Jamestown, N. Y. Ross G. Loop, '97, 3d Vice-Pres., Elmira, N. Y. Henry A. Stadlinger, '97, 4th Vice-Pres., Buffalo, N. Y. M. Louise Hurrell, '02, 5th Vice-Pres., Rochester, N. Y. Julius Richter, '04, Secretary, Buffalo, N. Y. Frank E. Brundage, '09, Treasurer, Buffalo, N. Y.

Representative of the Medical Alumni on University Council, Peter W. Van Peyma, '82, Buffalo, N. Y.

Trustees: Charles L. Preisch, '98, Chairman, Lockport, N. Y. Henry C. Buswell, '88, Buffalo, N. Y. Grover W. Wende, '89, Buffalo, N. Y. George F. Cott, '84, Buffalo, N. Y. Lesser Kauffman, Secretary, Buffalo, N. Y.

Executive Committee: William F. Jacobs, '08, Chairman, Buffalo, N. Y. Harry R. Trick, '01, Buffalo, N. Y. William G. Bissell, '92, Buffalo, N. Y. The President, Secretary and Treasurer, ex-officio.

Reception Committee: Fredolin Thoma, '87, Chairman; Edith R. Hatch, '06, Vice-Chairman; H. R. Hopkins, '67; Francis E. Fronczak, '97; Robt. Hebenstreit, '72; Augustus W. Hengerer, '02; Burt Hoyer, '82; John Tinkler, '07; Nellie Victoria Chappell, '92; John Kohlhas, '12.

The officers elected for the ensuing year are as follows: President, Wm. G. Bissell, '92, Buffalo; First Vice-President, Jacob Frank, '82, Chicago; Second Vice-President, Geo. E. Blackham, '70, Dunkirk; Third Vice-President, H. J. Knickerbocker, '98, Geneva; Fourth Vice-President, Edith R. Hatch, '06, Buffalo; Fifth Vice-President, John A. Conway, '03, Hornell; Secretary, A. H. Aaron, '12, Buffalo; Treasurer, James Sullivan, '10, Buffalo; Representative of Medical Alumni on Council, Grover W. Wende, '89, Buffalo; Trustee, Walter D. Greene, '76, Buffalo; Executive Committee, Chairman, Wm. F. Jacobs, '08, Buffalo; N. L. Burnham, '96, Buffalo; Harry Weed, '03, Buffalo.

Thursday, May 31st. "The Nature and Theory of Fatigue." F. H. Pratt, A. M., M. D., Prof. of Physiology, Buffalo, N. Y. "Some Studies in Nephritis." Richard N. DeNiord, M. D.,

'15, Buffalo, N. Y. "Practical Experiences with Radium." Dr. J. A. P. Millet, N. Y. State Institute for Malignant Diseases, Buffalo. "Soil Pollution." Victor G. Heiser, M. D., Director for the East, The Rockefeller Foundation, International Health Board, New York City. Evening. Class Reunions. Classes of '67, '72, '77, '82, '87, '92, '97, '02, '07, '12, will hold their reunions and dinners as arranged by the respective class committees.

Friday, June 1st. "The Relation of the Internal Secretions to Gynecology." Samuel W. Bandler, M. D., Prof. Diseases of Women, N. Y. Post Graduate Medical School and Hospital, New York City. "Two Years on the French Front." Illustrated with Lantern Slides, by our fellow alumnus, John Hanavan, M. D., '06, East Aurora, N. Y. Luncheon in College Library. Annual Business Meeting. Election of Officers and other routine business. Gynecology Diagnostic Clinic. Professor Bandler. "Some Practical Considerations in Cardiac Conditions." Arthur J. Patek, M. D., Associate Professor of Medicine, Marquette University, Milwaukee, Wis. "Some Phases of War Time Service in U. S. A." Illustrated with Lantern Slide and Motion Picture films. J. I. Mabee, M. D., Major Medical Corps, U. S. A. (Major Mabee absent on military duty). Fraternity Reunions. The College Fraternities, Nu Sigma Nu, Phi Rho Sigma, Omega Upsilon Phi, will entertain their Alumni at their Chapter Houses.

Saturday, June 2d. "Immunity in Syphilis." Hans Zinsser, M. D., New York, Prof. of Bacteriology, College of Physicians and Surgeons, Columbia University. "A Critical Consideration of Some of the Present Problems in Syphilis." Wm. Allen Pusey, M. D., Professor of Dermatology, University of Illinois, Chicago, Ill. "Cultural Limitations and the Neurasthenic Patient." Robert T. Morris, M. D., Professor of Surgery, Post Graduate Medical School and Hospital, New York City. Luncheon in College Library. "Typhus Fever Vaccination." With experiences in Serbian Epidemic in 1916. Harry Plotz, M. D., Mt. Sinai Hospital Staff, New York City. Diptheroid Infections with Special Reference to Hodgkins' Disease, Dr. R. R. Mellon, Pathologist, Hahnemann Hospital, Rochester. Annual Alumni Dinner. Banquet Hall Hotel Statler. Delancey Rochester, M. D., '84, Toastmaster.

---

**The Medical Society of the County of Erie** held a special meeting, June 7, in Alumni Hall, University of Buffalo, to recruit for the Medical Reserve Corps. Lt. Col. A. H. Briggs, M. D., a veteran of the Civil and Spanish Wars, spoke of the importance of disease which until the present war has killed more men than strictly military casualties. He also referred to the unpreparedness at the time of the Spanish-American War. He stated that he had kept the check sent him for final payment of services during that war and would invest the entire amount in Liberty bonds. Capt. B. J. Lee, 1st Lt. J. B. Clark of N. Y. and Capt. Herbert A. Smith of Buffalo, represented the Medical Reserve Corps and described the duties and pay of officers. It was stated that about a quarter of the entire profession of the country would be needed and that the quota for Buffalo would be about 200. (Owing to the necessity of leaving enough physicians to care for the civil population, it is obvious that the cities will be drawn upon to a greater degree than the country). To date, no where near the number of volunteers have been received from Buffalo, whereas many cities have complied fully with the demand. Curiously enough, the older men have volunteered in larger numbers, proportionately, than the younger. It may not be out of place to call attention to the fact that physicians of military age are subject to draft and that, at present, there is no system of drafting officers so that, as has been pointed out to some reluctant to volunteer as surgeons, the draft means service as a private. An interesting sidelight was thrown on domestic and social economics in the U. S. when Capt. Smith warned men unwilling to abandon lucrative private practices that these might dwindle and remarked "Consider the value, then of a fixed pay of $2400 a year (for Captain), $2,000 of which you can send home to the family." Probably this is no exaggeration of the average generosity of American heads of family (titular). Reserve officers from this part of the country will probably be sent to Ft. Benjamin Harrison for training.

---

**The United Physicians of Buffalo.** On May 24, a large meeting of physicians organized an association to include all members of the profession of Buffalo to last during the war. The following plan of organization was unanimously adopted:

The constitution is as follows:

The name of this organization shall be The United Physicians of Buffalo.

Its objects shall be to render patriotic service to the government in every way open to the profession; to provide care and treatment for sick and wounded soldiers and sailors; to care for the practices of doctors enlisted or drafted for military duty and their families, if necessary; to meet emergencies affecting the medical profession which may arise during the war, and to act in co-operation with other civic, patriotic or philanthropic organizations or committees.

Its membership shall be open to all physicians of Buffalo.

Its officers shall be a President, a Vice-President, a Secretary, a Treasurer, and an executive committee of seven members. The elected officers shall be ex-officio members of the Executive Committee. The officers shall be elected by this organization. The Executive Committee shall be appointed by the President. The officers first elected shall hold office for the remainder of the year 1917, or until their successors are elected. Thereafter the term of office of the elected officers shall be one year, or until their successors are elected.

Needed funds shall be raised by assessment voted by the membership of the organization.

The Executive Committee may appoint an Advisory Committee which shall represent all elements of the profession.

The officers elected at this meeting were: President, Dr. John H. Pryor; Vice-President, Dr. Arthur W. Hurd; Secretary, Dr. George A. Sloan; Treasurer, Dr. George W. Critchlow.

The president appointed the following physicians to serve on the Executive Committee: Dr. James W. Putman, Dr. Francis M. O'Gorman, Dr. Edward J. Meyer, Dr. Earl P. Lothrop, Dr. Charles S. Jewett, Dr. F. Park Lewis, Dr. Irving W. Potter.

Officers are ex-officio members of the Executive Committee and hold office for one year. The Executive Committee have appointed an Advisory Committee of about 60 physicians representing all elements in the profession: Dr. B. Bartow, Dr. A. G. Bennett, Dr. E. G. Bodenbender, Dr. C. R. Borzilleri, Dr. E. A. Bowerman, Dr. A. H. Briggs, Dr. J. Burke, Dr. H. C. Buswell, Dr. A. D. Carpenter, Dr. F. J. Carr, Dr. A. H. Cook, Dr. L. M. Francis, Dr. F. E. Fronczak, Dr. E. L. Frost, Dr. J. P. Gimbrone, Dr. W. S. Goodale, Dr. M. Hartwig, Dr. H. E. Hayd, Dr. L. Hendee, Dr. G. A. Himmelsbach, Dr. H. R. Hopkins, Dr. S. Y. Howell, Dr. L. Rowe, Dr. B. C. Johnson, Dr. L. Kauffman, Dr. C. N. Kendrick, Dr. J. E. King, Dr. P. LeBreton, Dr. J. H. Lewis, Dr. J. S. Lewis, Dr. E. H. Long, Dr. M. D. Mann, Dr. W. H. Mansperger, Dr. B. C. Maycock, Dr. W. H. Marcy, Dr. J. A. McLeod, Dr. T. H. McKee, Dr. E. R. McGuire, Dr. J. G. Meidenbauer, Dr. F. A. Mendlein, Dr. W. M. Mehl, Dr. J. J. Mooney, Dr. G. T. Mosely, Dr. F. J. Parmenter, Dr. J. H. Potter, Dr. W. S. Renner, Dr. L. Reu, Dr. De L. Rochester, Dr. H. C. Rooth, Dr. R. M. Schley, Dr. DeW. H. Sherman, Dr. I. M. Snow, Dr. Herbert A. Smith, Dr. C. G. Stockton, Dr. J. C. Thompson,

Dr. E. S. Tobie, Dr. P. W. VanPeyma, Dr. T. J. Walsh, Dr. A. L. Weil, Dr. G. W. Wende, Dr. A. E. Woehnert, Dr. J. V. Woodruff, Dr. J. F. Whitewell, Dr. G. W. York.

The following plan was presented for consideration at a meeting at the Hutchinson High School, June 12th.

As soon as a physician knows the date of expected departure for military service he should send a notification to his patients, stating the possible duration of his absence and the names of the physicians he recommends to be called in case of illness.

He may make any arrangements he pleases, financially or otherwise, with the physician selected to care for his practice.

When other physicians not designated by him are consulted they should inquire who has been the attending physician, and if the physician named is absent on military duty all fees received for services rendered in substitution must be delivered to the family of the absent physician, or to him personally upon his return.

The amount of the collection should be recorded with the name of the patient or family making the payment.

Should it come to the knowledge of the physician who has been absent on military duty that this agreement has been violated a complaint with the facts should be filed with the Secretary, who will present the matter to the Executive Committee for consideration and decision.

Every reasonable effort should be made to secure retention of any appointments held by the physician during the period of his military service. The vacancy should be filled temporarily during his absence.

If the physician at any time during military service decides to remain permanently in government service and relinquish local practice and residence he should promptly notify the Secretary and those selected to assume his practice.

The Executive Committee suggests a committee be appointed to collect outstanding accounts of those physicians who enter service at a cost of 10%.

It is agreed that no members of this organization will render medical service to any patient or family or continue in attendance upon the same after the physician has returned from service and resume practice.

### SUGGESTED FORM FOR NOTIFICATION TO PATIENTS.

M..............................

..............................

Dear..............................

As a member of the United States (Army-Navy) I have been ordered into active service by the Government, and on that account I am writing you, so that, in case of illness, you may summon

Dr. .............................. of....................

Telephone No................., who has kindly consented to attend my patients in my absence. I can heartily recommend him.

Sincerely,

In case of any illness, trouble or emergency, financial or otherwise, affecting yourself or any dependent, communicate, or have others do so, with the Secretary, whose name and address you should carefully preserve.

If any dependent, particularly wife or child, is affected by illness the physician in attendance should report when a trained nurse, hospital care or special service is required if financial circumstances will not permit of this expenditure. Any assistance rendered to dependents will constitute an act of simple justice. It will be rendered in secret.

---

The second convocation of the **American College of Physicians** occurred on Tuesday, June 5th, 1917, at the Hotel Nassau, Long Beach, Long Island, N. Y. About seventy eight of the members were present. The impressive ceremony was presided over by the President, Dr. Reynold Webb Wilcox.

About forty candidates in academic costume were introduced with the appropriate words by the Secretary-General, Dr. Heinrich Stern, to the President, who handed them their certificate of election to Fellowship. The American College of Physicians now consists of about one hundred and ten Fellows.

In order to be considered eligible for Fellowship in the American College of Physicians by the Council of the College it is necessary that a candidate be a member of the American Congress on Internal Medicine; be a Doctor of Medicine for ten years, a teacher of medicine and laboratory investigator of wide clinical experience. He must have published either frequent contributions to medical periodicals or noteworthy medical literature.

The next convocation will take place in Pittsburgh during Christmas week, together with the meeting of the American Congress on Internal Medicine and the American Association for the Advancement of Science, with which both organizations are affiliated.

